# HOX Genes Family and Cancer: A Novel Role for Homeobox B9 in the Resistance to Anti-Angiogenic Therapies

**DOI:** 10.3390/cancers12113299

**Published:** 2020-11-08

**Authors:** Serena Contarelli, Vita Fedele, Davide Melisi

**Affiliations:** Digestive Molecular Clinical Oncology Research Unit, Section of Medical Oncology, Department of Medicine, University of Verona, 37134 Verona, Italy; serena.contarelli@univr.it (S.C.); vita.fedele@univr.it (V.F.)

**Keywords:** angiogenesis, anti-angiogenic therapy, therapeutic resistance, HOXB9

## Abstract

**Simple Summary:**

The inhibition of angiogenesis, relying on the use of drugs targeting the VEGF signaling pathway, has become one of the main strategies for cancer treatment. However, the intrinsic and acquired resistance to this type of therapy limit its efficacy. Thus, the identification of novel therapeutic targets is urgently needed. The resistance to anti-angiogenic treatment often occurs through the activation of alternative VEGF independent signaling pathways and recruitment of bone marrow-derived pro-angiogenic cells in the tumor microenvironment. HOX genes are key regulators of embryonic development, also involved in angiogenesis and in cancer progression. HOXB9 upregulation occurs in many types of cancer and it has been identified as a critical transcription factor involved in tumour resistance to anti-angiogenic drugs. Indeed, HOXB9 modulates the expression of alternative pro-angiogenic secreted factors in the tumour microenvironment leading tumor escape from the anti-angiogenic treatments. Hence, HOXB9 could serves as a novel therapeutic target to overcome the resistance to anti-angiogenic therapies.

**Abstract:**

Angiogenesis is one of the hallmarks of cancer, and the inhibition of pro-angiogenic factors and or their receptors has become a primary strategy for cancer therapy. However, despite promising results in preclinical studies, the majority of patients either do not respond to these treatments or, after an initial period of response, they develop resistance to anti-angiogenic agents. Thus, the identification of a novel therapeutic target is urgently needed. Multiple mechanisms of resistance to anti-angiogenic therapy have been identified, including the upregulation of alternative angiogenic pathways and the recruitment of pro-angiogenic myeloid cells in the tumor microenvironment. Homeobox containing (HOX) genes are master regulators of embryonic development playing a pivotal role during both embryonic vasculogenesis and pathological angiogenesis in adults. The importance of HOX genes during cancer progression has been reported in many studies. In this review we will give a brief description of the HOX genes and their involvement in angiogenesis and cancer, with particular emphasis on HOXB9 as a possible novel target for anti-angiogenic therapy. HOXB9 upregulation has been reported in many types of cancers and it has been identified as a critical transcription factor involved in resistance to anti-angiogenic drugs.

## 1. Introduction

Angiogenesis is a highly regulated physiological process, consisting in new blood vessels formation from preexisting ones, which exerts a crucial role during embryonic development and wound healing process in adults. Angiogenesis mis-regulation can contribute to the development of various disorders, including cancers [1], and it is often triggered by low tissue oxygen concentrations. Low oxygen levels induce the expression of several different growth factors and pro-angiogenic factors, including vascular endothelial growth factor (VEGF), angiopoietins (ANGs), fibroblast growth factors (FGFs), transforming growth factors (TGFs), and placental growth factor (PlGF), which play essential roles in cancer development. 

The identification of VEGF as master regulator of the angiogenic process [2] has led to the development of several new therapeutic agents targeting the VEGF-signaling pathway, including monoclonal antibodies, recombinant proteins, and small tyrosine kinase inhibitors (TKI) [3] (Table 1). Bevacizumab and Ramucirumab are among the monoclonal antibodies targeting the VEGF-VEGFR2 signaling pathways that have been approved for the treatment of solid cancer [4]. Bevacizumab has been approved in 2004 by the US Food and Drug Administration (FDA) for the treatment of metastatic colorectal cancer [5], while Ramucirumab has been approved for the treatment of gastric adenocarcinoma, metastatic non-small cell lung carcinoma and colorectal cancers. [6,7]. Aflibercept is among the recombinant proteins targeting the VEGF signaling pathway; Aflibercept is a soluble recombinant VEGFR fusion protein that inhibits multiple VEGF family members (i.e., VEGFA, VEGFB, and PIGF) which has been approved for the treatment of metastatic colorectal cancer [8]. 

Despite the promising results from preclinical experiment, blocking the VEGF signaling pathway appears to be ineffective in certain type of cancers or in certain groups of patients [9]. After an initial period of clinical benefit, patients develop resistance to anti-angiogenic therapeutic drugs, associated with a rapid boost of angiogenesis and tumor growth. The resistance to anti-angiogenic treatment often occurs through the activation of alternative VEGF-independent signaling pathways such as recruitment of bone marrow-derived pro-angiogenic cells in the tumor microenvironment and/or tumor cells reprogramming into a more aggressive phenotype [10].

Homeobox genes play a central role in regulating neovascularization during both embryonic vasculogenesis and pathological angiogenesis in adults. Recent studies have identified Homeobox B9 (HOXB9) as a crucial transcription factor involved in tumor resistance to anti-angiogenic drugs. HOXB9 is upregulated in many type of cancers [11,12,13] and has been proposed as a factor modulating the expression of alternative proinflammatory and pro-angiogenic secreted factors in the tumor microenvironment [14]. Therefore, targeting HOXB9 could represent a good therapeutic approach to overcome the resistance to anti-angiogenic therapies.

## 2. HOX Family Transcription Factors

**Figure 1:**
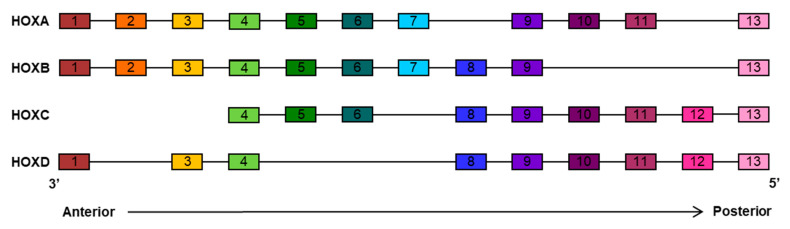
Arrangement of the mammalian HOX clusters. Thirty-nine *HOX* genes are divided into four separate clusters (HOXA, HOXB, HOXC, and HOXD) located on four distinct chromosomes. During embryonic development, *HOX* genes are activated in a 3’ to 5’ manner within each cluster, which is relevant for the temporal and spatial gene activation concomitant with the development of the antero-posterior axis. *HOX* genes with the same number are referred to as paralogs.

*HOX* genes encode for a highly evolutionarily conserved family of homeodomain-containing transcription factors known to be key regulators of embryonic development. The homeobox genes, originally discovered in *Drosophila melanogaster* [15,16], are present in organisms ranging from primitive chordate to humans. During vertebrate evolution, the ancestral *HOX* gene cluster has been repeatedly duplicated to generate groups of paralogue genes sharing high similarities in sequence, expression pattern, and function. In mammals, the *HOX* family gene contains 39 genes organized into four clusters, called HOXA, HOXB, HOXC, and HOXD, which are located in four different chromosomes (7p15, 17p21, 12q13, and 2q3 respectively). The 39 genes have been divided into 13 paralog groups on the basis of sequence similarity and chromosomal position within each linkage group. Each cluster contains 9 to 11 members [17] (Figure 1). 

Each mammal *HOX* gene contains two exons and a single intron. *HOX* genes encode for transcription factors containing a homeodomain of approximately 180 bp located in their second exon. The homeodomain encodes for a highly conserved DNA-binding domain containing a helix-turn-helix motif of 60 amino acids responsible for recognition and binding of their target gene promoters [18,19]. HOX proteins bind DNA cooperatively with members of the three-amino-acid loop extension (TALE) protein family as cofactors to drive the transcription of downstream targets [20]. 

The genes of each *HOX* clusters are arranged along the chromosome in a sequence reflecting their order of expression during embryogenesis. From the gastrula stage onward, the activation of these genes occurs sequentially according to their position within each clusters, in the sense that *HOX* genes located at the 3’ ends (paralog group 1) are expressed earlier and more anteriorly than those located at the 5’ ends (paralog group 13) [21]. This property is referred to as spatial and temporal collinearity. Although the expression pattern progressively changes between adjacent paralog groups, members of the same paralog groups often exhibit similar expression and protein functions. Genetic studies have shown that some of these *HOX* genes work together, highlighting the existence of a functional redundancy among *HOX* paralogous genes. Hence, a defect in one gene can be compensated by the activity of another one [22,23].

Although *HOX* genes are master regulators of embryo development, they are also required for proper functioning of adult tissues, controlling cellular identity and regulating numerous processes including proliferation, apoptosis, differentiation, motility, and angiogenesis [24,25,26,27]. A detailed discussion about the role of *HOX* genes in proliferation, motility, apoptosis, and differentiation is outside the scope of this review. The role and involvement of *HOX* genes in angiogenesis will be briefly discussed below, before moving on to a more detailed discussion about the role of HOXB9 in cancer development and angiogenesis and its possible role as a target for anti-angiogenic therapy.

### 2.1. HOX Family in Angiogenesis

HOX proteins can have stimulatory or inhibitory effects on angiogenesis. The strongest evidence of the involvement of *HOX* genes in regulating endothelial cells (ECs) phenotype comes from studies conducted on the paralog group HOX3. In particular, HOXA3, HOXB3, and HOXD3 have been reported to be positive regulators of angiogenesis. HOXD3 is highly expressed in active proliferating ECs forming tubes but not in quiescent ECs, and its expression is induced by bFGF [28]. HOXD3 overexpression is associated with the invasive phase of angiogenesis [28]. HOXD3 enhances the expression of integrin α5 and β3 subunits and upregulates the expression of serine protease urokinase-type plasminogen activator (uPA). The aforementioned factors, integrin α5, β3 subunits and uPA, are poorly expressed in quiescent endothelium; however, their expression increases in response to angiogenic stimuli and is essential for ECs adhesion, migration, and invasion [29]. Inhibition of HOXD3 expression significantly blocks the ability of bFGF to induce the expression of both α5 and β3 integrin subunits and uPA, and this is sufficient to inhibit angiogenesis. HOXD3 overexpression, on the other hand, leads to an increased expression of these two proteins, along with morphological ECs changes [28]. 

HOXA3 expression correlates with an increased ECs migration. The effect exerted on migration by HOXA3 is given by the upregulation of genes involved in cell–cell interactions or cell–extracellular matrix interaction (e.g., matrix metalloproteinase 14). Knockout mice models have also shown that disruption of *HOXA3* gene results in cardiovascular defects. During embryonic development, the loss of this gene affects the intrinsic ability of the neural crest cells to induce proper differentiation of the third pharyngeal pouch. The neural crest cells within the third pharyngeal arch, precursor of the carotid artery, initially migrate properly but arteries become sinusoidal and degenerate at the time of differentiation, and have a decreased rate of proliferation [30]. 

The mechanism by which the expression of HOXB3 influences angiogenesis is different compared to HOXD3 and it has been associated with capillary morphogenesis in endothelial sprouts. Blocking HOXB3 expression markedly reduces the expression of the angiogenic ligand ephrin A1 and impairs capillary morphogenesis of dermal microvascular ECs, whereas constitutive expression of HOXB3 results in an increase in capillary vascular density and angiogenesis [31]. Taken together, these observations suggest a complementary function of the *HOX* genes paralogous, resulting in HOXD3 promoting the invasion and/or migration of ECs, in response to angiogenic signals, and HOXB3 promoting the subsequent capillary morphogenesis of the newly formed vascular sprouts.

HOXA9 is also involved in angiogenesis regulation, its overexpression promotes ECs migration and tube formation in vitro through an upregulation of the Ephrin receptor B4 (EPHB4) [32]. HOXA9 acts as a master regulator of endothelial committed genes and is able to upregulate the endothelial nitric oxide synthase (eNOS) and the VEGFR2. Consistently, HOXA9-deficient mice display a decreased number of EC precursor cells and show an impaired response to angiogenic stimuli [33]. 

Overexpression of HOXB5 leads to the induction of the VEGF receptor flk-1 and increases the number of platelet-endothelial cell adhesion markers in ECs. HOXB5 mRNA co-localizes with flk1 and activates cell-intrinsic events regulating the differentiation of angioblasts and mature ECs from their mesoderm-derived precursors [34]. 

The HOXB7 gene has been found to be constitutively expressed in primary melanoma tumors and their metastases, as well as in 25 melanoma cell lines. It has been reported to increase melanoma cells proliferation via upregulation of bFGF expression [35]. HOXB7 overexpression has also been associated with enhanced expression of several angiogenic growth factors including VEGF, Ang-2, and interleukin (IL)-8 in the breast cancer cell line SkBr3, indicating that HOXB7 is a critical factor for upstream pro-angiogenic genes. Consistently, tumors explanted from SkBr3-HOXB7 xenografts mice revealed greater levels of vascularization compared to their control counterparts [36]. 

Overexpression of HOXC10 has been reported to be able to enhance the ability of glioma cells to induce tube formation, migration, and proliferation of ECs, whereas silencing HOXC10 exerts the opposite effect. HOXC10 transcriptionally upregulates VEGFA expression directly binding its promoter [37]. 

Not all members of the HOX family have an angiogenesis-promoting role. HOXA5, paralog of HOXB5, is considered an anti-angiogenic gene and it is expressed in normal quiescent endothelium, but not in activated vasculature. HOXA5 expression is able to block angiogenesis in vivo and cell migration in vitro via downregulation of many pro-angiogenic genes, such as VEGFR2, ephrin A1, hypoxia-inducible factor (HIF)-1α and cyclooxygenase (COX) 2 and upregulation of the anti-angiogenic factor thrombospondin 2 [38]. Moreover, HOXA5 stabilizes adherens junctions, through β-catenin retention, and enhances Akt activity via phosphatase and tensin homolog (PTEN) downregulation resulting in the maintenance of a stable quiescent vascular phenotype [39].

HOXD10 is an anti-angiogenic gene as well; it is highly expressed in normal quiescent vascular endothelium, it impairs ECs migration and blocks angiogenesis induced by bFGF and VEGFA [40]. Studies conducted in both breast and endometrial cancer have shown a progressive reduction in HOXD10 expression as malignancy increases. In line with this observation, restoration of HOXD10 expression in breast cancer cells reduces migration and restores cell polarity with induction of acinar structure formation. This reverted phenotype is associated with downregulation of α3 integrin expression [41]. HOXD10 also attenuates tumor angiogenesis via downregulation of angiogenic factors including VEGFA [42]. 

## 3. HOXB9 in Cancer

HOXB9 is a transcription factor member of the well conserved HOXB cluster genes. HOXB9 is involved in the formation of the thoracic skeletal elements and contributes to forelimb development [43,44]. In adults, it is involved in mammary gland development during pregnancy [45] and in blood cell differentiation [46]. 

In addition to its crucial roles in development, HOXB9 plays an important role in numerous human solid cancers and its aberrant expression significantly contributes to tumor formation [47,48] (Table 2). High levels of HOXB9 are associated with poor prognosis in lung adenocarcinoma patients [12], low overall survival of colon cancer patients [49], high tumor grade and lower overall survival of breast cancer patients [50], advanced clinical stage of glioma patients [51], tumor progression, vascular and lymphatic invasion in gastric cancer patients [52], and vascular invasion and poor overall survival of hepatocellular carcinoma patients [13]. However, it has also been reported that downregulation of HOXB9 is associated with poor survival of gastric carcinoma patients, highlighting conflicting hypotheses about HOXB9’s role in cancer [53].

### 3.1. HOXB9 Regulation in Cancer

Numerous recent studies have underlined the central role of HOXB9 in promoting cancer progression, metastasis, and resistance to anti-angiogenic treatments.

A variety of mechanisms have been proposed to be responsible for the mis-regulation of HOXB9 during cancer progression. HOXB9 is a downstream target of WNT/transcription factor 4 (TCF4). The Wnt signaling pathway controls self-renewal of numerous tissues and plays a critical role in oncogenesis throughout the regulation of genes involved in cell proliferation, survival, and metastasis [69]. The activation of the WNT/TCF4-HOXB9 signaling pathway enhances the ability of human lung adenocarcinomas to develop brain and bone metastases. Accordingly, short hairpin RNA (shRNA)-mediated knockdown of HOXB9 in lung cancer cells reduces their invasive phenotype [63]. HOXB9 expression is also induced by N-acetyl-galactosaminyl-transferases 14 (GalNAc-T14). GalNAc-T14 increases the stability of β-catenin and the stability of the downstream Wnt pathway target genes, including HOXB9. A meta-analysis conducted on clinical genomic data revealed that the expression of GalNAc-T14 or HOXB9 strongly correlated with reduced recurrence-free survival and increased hazard risk in patients with lung adenocarcinoma, indicating a possible clinical relevance and their involvement in metastasis [64].

*HOX* genes are critical developmental regulators and growing evidence have identified sex steroids as regulators of their expression in mature tissues [70]. In this regard, several studies have reported that HOXB9 promoter contains multiple estrogen-response elements, demonstrating that HOXB9 gene is transcriptionally regulated by estrogen [71]. Other studies identified E2F transcription factors, a family of transcription factors involved in the cell cycle regulation, as direct regulator of HOXB9 expression. In particular, E2F1 was reported to bind the binding site of the HOXB9 promoter. The induction of HOXB9 expression by E2F1 was observed in several breast cancer cell lines and a significant correlation between E2F1 and HOXB9 was revealed in clinical breast cancer samples indicating their potential role in breast cancer progression [56]. 

Recent studies have suggested that post-translational modifications, such as acetylation, might have a possible role on HOXB9 regulation. The acetylated form of HOXB9 decreases the ability of lung cancer cells to migrate and to promote tumor growth in mice. Furthermore, HOXB9 acetylation at K27 predicts a better prognosis for patients with lung adenocarcinoma [58]. The importance of the acetylated and non-acethylated state of HOXB9 has also been described in colon and pancreatic cancer [67,72]. Colon cancer patients having low levels of the acetylated HOXB9 form have a more favorable outcome compared to patients with high levels of the non-acetylated HOXB9 form [72]. The non-acetylated HOXB9 form promotes the transcription of downstream *JMJD6* and *EZH2* target genes, whereas the acetylated HOXB9 form is translocated into the cytoplasm, and thereby cannot longer function as transcription factor [72]. 

## 4. The Role of HOXB9 in Tumor Anti-Angiogenic Treatments Escape

**Figure 2:**
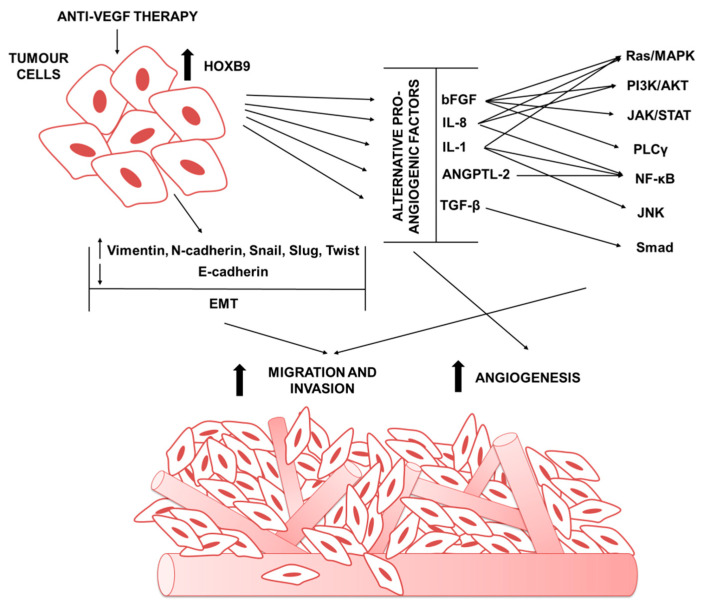
The involvement of HOXB9 in tumor resistance to anti-angiogenic drugs. HOXB9-mediated angiogenesis correlates with increased expression of alternative proinflammatory and pro-angiogenic secreted factors in the tumor microenvironment, including vascular endothelial growth factor (VEGF), basic fibroblast growth factor (bFGF), Angiopoietin-like protein 2 (Angptl2), transforming growth factor beta (TGF-β), interleukin (IL)-1, and interleukin (IL)-8, which initiate an extensive angiogenic program enabling tumor vascularization. HOXB9 overexpression is associated with a transition from an epithelial phenotype into a more mesenchymal phenotype (EMT) by reduction in E-cadherin expression levels and increasing of mesenchymal markers expression, including vimentin, N-cadherin, and the transcription factors Snail, Slug, and Twist, leading to the ability of tumor cells to migrate out of the confine of the ducts and invade into the blood vessel, and migrate to distant site to initiate metastatic tumor growth.

The inhibition of angiogenesis by blocking pro-angiogenic factors and/or the activity of their receptors has become a primary strategy for cancer therapy, although the improvements provided by these therapies remain limited [9]. Indeed, after an initial period of clinical benefit [73], almost inevitably, tumors adapt and continue to grow, resulting in more aggressive tumors with an acquired resistance to anti-angiogenic treatment such as VEGF/VEGFR inhibition [74]. The acquisition of resistance to anti-angiogenic treatment occurs via activation and/or upregulation of alternative angiogenic pathways promoting tumor angiogenesis in a VEGF-independent manner and via the recruitment of pro-angiogenic myeloid cells in the tumor microenvironment [10,75]. A correlation between HOXB9 expression and the upregulation of a pro-angiogenic signaling cascade has indeed been observed, supporting the hypothesis that HOXB9 could be involved in the resistance to anti-angiogenic treatments (Figure 2).

### 4.1. HOXB9 in Mediating the Expression of Alternative Pro-Angiogenic Factors

Although the VEGFs protein family are key regulators of angiogenesis, the existence of alternative growth factors—including ANGs, FGFs, TGFs, PlGF, granulocyte colony-stimulating factor (G-CSF), stromal cell-derived factor-1 (SDF-1), and hepatocyte growth factor (HGF)—responsible for endothelial activation, vessel formation, and stabilization has become evident in several preclinical and clinical studies. Thus, targeting a single angiogenic growth factor has limited therapeutic effect. In this regard, many evidences from preclinical models and clinical trials have shown that inhibition of a specific growth factor can induce the expression of alternative proinflammatory and pro-angiogenic secreted factors in the tumor microenvironment. For instance, bevacizumab resistance metastatic colorectal cancer shows an upregulation of HIF-1α [76]. Indeed, increased hypoxia leads to an upregulation of other pro-angiogenic factors, thereby bypassing the VEGF-dependent angiogenesis [75].

Although not an oncogene, HOXB9 expression has been reported to promote neovascularization and distal metastasis, suggesting that the aberrant overexpression of HOXB9 contributes to cancer progression and invasiveness. HOXB9 has an effect on angiogenesis in many different types of cancer cells, including colon [57], ovarian, renal [27], breast [50,77], hepatocellular [13], and prostate [68]. It has been reported that HOXB9-mediated angiogenesis correlates with increased concentrations of several growth factors, including VEGF, bFGF, Angiopoietin-like protein 2 (Angptl2), TGF-β, interleukin (IL)-1 and IL-8—which are involved in proliferation and differentiation of ECs—regulation of vascular permeability, and cell–matrix interactions [78]. 

The FGF family of growth factors comprises a series of important and potent mediator of tumor angiogenesis. The mammalian FGF family comprises 22 members, 18 of which are secreted proteins that interact with 4 FGF tyrosine kinase receptors (FGFRs) and 4 intracellular non-signaling proteins that serve as cofactors for voltage gated sodium channels [79]. The FGF signaling has a fundamental role in developmental pathways, including embryogenesis and organogenesis, and in the adult, where it is important for tissue maintenance and tissue repair and regeneration [79,80]. FGF binding to its receptor FGFR tyrosine kinase leads to the activation of the RAS-MAPK, PI3K-AKT, PLCγ, and STAT intracellular signaling pathways [81]. The FGF signaling has a crucial role in tumor angiogenesis in stimulating new vessel formation and vessel maturation by driving ECs proliferation, promoting extracellular matrix degradation, and altering cell–cell adhesion receptors [82]. The FGF system can mediate resistance to anti-VEGFR therapeutic agents, and this was revealed by a preclinical genetically engineered mouse model of pancreatic islet carcinoma, the *Rip1-Tag2* mice. Prolonged treatment of *Rip1-Tag2* mice with a monoclonal antibody, the DC101, which specifically blocks VEGFR2 signaling, induces an initial transitory response, characterized by tumor stasis and reduction in tumor vascularity, followed by tumor regrowth with restoration of high blood vessel density. Analysis of the revascularized VEGFR2-blocked tumors revealed an increased expression of other pro-angiogenic factors, including FGFs and Ephrins, that re-stimulates tumor angiogenesis in a VEGF independent manner. When these mice were treated with the VEGFR inhibitor in combination with an FGF-trap, an attenuation of the revascularization and deceleration of tumor growth was observed, demonstrating a role of the FGF signaling in regulating angiogenesis [83]. A similar increased expression of bFGF along with other angiogenic factors, including PlGF and HGF, has been observed before disease progression in metastatic colorectal cancer patients treated with a combination of FOLFIRI and bevacizumab [84]. An analogous observation has come from a clinical study conducted on glioblastoma patients treated with a pan-VEGF receptor tyrosine kinase inhibitor, cediranib. In this study, an initial response of vascular normalization was observed, followed by a progression phase that was correlated with a significant increase in bFGF levels in the blood of relapsing patients, indicating an adaptive mechanism involving FGF signaling in anti-VEGF treatments [85]. 

Expression levels of inflammatory factors have also been linked to resistance to anti-VEGF therapy. For instance, increased IL-8 expression levels have been reported in patients with pancreatic and colorectal cancer resistant to VEGF-therapy and it has been linked to the recruitment of immunosuppressive myeloid cells [86]. IL-8 is a pro-inflammatory factor belonging to CXC chemokine family; IL-8, mainly produced by tumor cells, upon binding to its cell-surface receptors, CXCR1 (IL-8RA) and CXCXR2 (IL-8RB), can promote angiogenesis, survival of cancer stem cells, and recruitment of myeloid-derived suppressor cells [87]. IL-8 is a circulating factor regulated by the activation of TAK1 pathway and it is the most significant predictive biomarker of resistance to nal-IRI in patients with gemcitabine-refractory pancreatic cancer [88]. Increased levels of IL-8 expression have been reported in a head and neck squamous cell carcinoma tumor model resistant to anti-VEGF therapy [89], and in a clinical study conducted in patients with renal cell carcinoma treated with sunitinib [90]. Plasma levels of IL-8 are associated with poor outcomes in newly diagnosed glioblastoma patients treated with a pan-VEGF cediranib [91]. Moreover, IL-8 plasma levels could serve as a biomarker for resistance to sunitinib in patients with renal cell carcinoma [90]. 

IL-1α and IL-1β are proinflammatory cytokines that initiate and sustain the angiogenic process [92]. Recent studies conducted in murine pancreatic cancer models have identified the upregulation of both IL-1α and IL-1β as main players in sustaining resistance to anti-angiogenic therapy [86]. Both IL-1α and IL-1β bind to the type 1 IL-1 receptor (IL-1R1) leading to the recruitment of its co-receptor IL-1 receptor accessory protein (IL-1RAP) that is necessary for the activation of their downstream pathways, such as NF-κB, JNK and MAPK signaling cascade [93]. The autocrine secretion of IL-1Ra, a physiological inhibitor of IL-1R1, has been proven to be responsible for the constitutive activation of the NF-κB pathway [94,95,96]. Indeed, Anakinra, an FDA-approved recombinant IL-1Ra, when given alone or in combination with gemcitabine, reduces tumor growth through inhibition of IL-1α-induced NF-κB activation [97]. IL-1β induces the production of several angiogenic factors, such as HIF-1α, VEGF, and C-X-C motif chemokine (CXC) ligand 2 (CXCL2), which promotes rapid tumor cells growth and neovascularization in in vivo mouse models [98]. IL-1α and IL-1β were reported to be upregulated in an anti-VEGF resistant pancreatic cancer cells compared to the anti-VEGF sensitive cells [86]. Consistently, the neutralization of IL-1 signaling, in combination with the inhibition of other proinflammatory signaling pathways, such as CXCR1/2 and TGF-β, abrogates resistance to anti-VEGF therapy resulting in a significant reduction in tumor burden and a significant increase in overall survival in a in vivo murine model of pancreatic cancer resistance to anti-VEGF therapy [99]. 

TGF-β family members are multifunctional cytokines acting on different type of cells, including ECs and inducing angiogenesis in vivo [100]. TGF-β exerts its function through binding to type II and type I serine/threonine kinase transmembrane receptors complex. The ligand binding results in recruitment and phosphorylation of receptor regulated Smad2/3 proteins that associate with the common mediator, Smad4. After activation and translocation into the nucleus, they work as transcription factors regulating the expression of specific target genes [101]. In the last decade, it has been demonstrated that the inhibition of TGF-β signaling is an effective strategy for the treatment of pancreatic cancer patients in combination with classic chemotherapeutic agents or immune checkpoint inhibitors [102,103,104,105,106,107,108,109]. In particular, activin receptor-like kinase 1 (ALK-1) is a type I receptor with restricted expression in vascular ECs mediating the critical role of TGF-β in angiogenesis. The TGF-β/ALK1 signaling induces Smad1/5 activation that has been shown to stimulate Ecs migration, proliferation, and tube formation [110]. The importance of TGF-β signaling in angiogenesis and vascular remodeling has been underlined by numerous in vivo studies that have shown how loss of TGF-β signaling components leads to embryonic lethality because of cardiovascular defects [111]. ALK1 knockout mice died at mid-gestation because of severe vascular abnormalities and angiogenesis defects [112]. Higher levels of TGF-β have been found in many tumor tissues compared to the adjacent normal tissues and its expression correlates with patient survival [113,114]. Many studies reported deregulation of the TGF-β pathways at different levels. High levels of TGF-β expression have been found in glioma models resistant to anti-VEGF therapy and in several other preclinical models, suggesting that it might play an important role in the acquired resistance to anti-angiogenic therapy [115,116]. Intriguingly, an upregulation of TGF-β has been reported in murine models resistant to the anti-VEGF antibody bevacizumab [86] and the inhibition of the TGF-β signaling abolished the resistance to anti-VEGF therapy leading to reduction in tumor burden and significant prolonged survival compared to mice treated with bevacizumab alone [99].

Angptl2 belongs to the angiopoietin-like family and is a tumor-promoting secreted glycoprotein [117]. Angptl2 exerts its function in tissue repair and vasculogenesis; however, excess Angptl2 signaling causes chronic inflammation and subsequent pathological tissue remodeling, leading to the development of different diseases, including cancers [118,119]. The autocrine signaling of Angptl2 and its receptor LILRB2 plays a key role in sustaining epithelial to mesenchymal transition (EMT) and the early metastatic events in pancreatic pre neoplastic lesions [120]. In transgenic mice models, overexpression of Angptl2 induces a significant increase in the number of blood vessels compared to Angptl1 expressing mice, suggesting that Angptl2 promotes angiogenesis in vivo [121]. Similarly, knocking down both Angptl1 and Angptl2 produces severe vascular defects partially due to increased apoptosis of ECs at the sprouting stage, indicating their central role in vascular development [122]. Recent studies have reported an upregulation of Angptl2 in a murine pancreatic cancer model resistant to anti-VEGF therapy, suggesting that it could play an important role in the resistance to anti-VEGF therapy [86]. 

Angiogenic factors induced by HOXB9 activation in breast cancer cells leads to microenvironment enrichment with angiogenic factors responsible for the formation of large highly vascularized tumors that metastasize to the lung in mouse xenograft model. Moreover, HOXB9 overexpressing breast cancer cells display a significant induction of new blood vessel formation in vivo, whereas HOXB9 downregulation leads to the suppression of new tube formation [11]. These findings have also been reported in prostate cancer, where HOXB9 downregulation inhibits the angiogenic process via reduction in HUVEC tube formation and HIF-1α and VEGF reduces expression [68], and in colorectal cancer where HOXB9 overexpressing xenograft mice showed an increase in tumor burden and micro vessel density [57]. The presence of multiple HOXB9 binding sites at promoters of these pro-angiogenic factors [54] suggests that these genes are likely to be targets of HOXB9 activation. 

A recent study has identified microRNA-192 as a key regulator of tumor angiogenesis in both highly angiogenic ovarian and renal cancer models. Disrupting the crosstalk between tumor and ECs by targeting two key transcription factors, EGR1 and HOXB9, leads to a global downregulation of genes involved in angiogenesis pathways. Consistently, nanoliposome-mediated delivery of microRNA-192 significantly inhibits tumor angiogenesis resulting in a much more profound anti-tumor effect compared to that observed with murine anti-VEGF antibody treatment, suggesting the central role of EGR1 and HOXB9 downstream target in tumor angiogenesis [27]. 

HOXB9, throughout regulation of pro-inflammatory and pro-angiogenic secreted factors expression, including Angptl2, CXCL1, IL-8, and TGF-β play a crucial role in sustaining resistance to angiogenic targeting therapy. Indeed, a recent study conducted in a resistant anti-angiogenic preclinical model has revealed that HOXB9 is a key transcription factor in sustaining tumor resistance to anti-VEGF treatments. The study reported that HOXB9-positive tumors were resistant to anti-VEGF therapy, whereas mice bearing HOXB9-negative tumors were cured by treatment with this drug. Accordingly, silencing HOXB9 in the anti-VEGF-resistant xenograft model significantly decreased the expression of the alternative secreted pro-angiogenic factors inducing sensitivity to the anti-angiogenic therapy and resulting in prolonged survival in vivo [14]. 

All together, these studies suggest an important role of others pro-angiogenic factors in the resistance to anti-angiogenic therapy and highlight HOXB9 as a crucial transcription factor in sustaining tumor resistance to anti-VEGF treatment [11,54]. 

### 4.2. HOXB9 Role in Tumor Invasivenes and Metastasis

There is increasing evidence supporting the idea that therapeutic inhibition of angiogenesis correlates with an increased local invasiveness and distant metastasis despite overall inhibition of tumor growth [123,124]. It has been reported that increased metastasis and enhanced invasiveness in response to anti-angiogenic therapy is variable, i.e., it is dependent on the tumor model, treatment type, dosing, and scheduling. Notably, it has been shown that high doses and short term anti-angiogenic treatments have the most deleterious effects, enhancing metastasis to distant organs and resulting in reduced survival [123,124].

Anti-angiogenic treatment can promote a more permissive metastatic potential, both in tumor and normal organ vessels. Angiogenesis targeting drugs lead to a disruption of the vasculature integrity via reduction in vascular basement membrane and pericyte coverage, increased leakiness and decreased adherens junction protein expression [125,126]. These vascular changes results in an increased intravasation and facilitates the passage of tumor cells into the circulatory system, thus facilitating extravasation and metastatic colonization of distant organs [126]. 

In addition, anti-angiogenic treatment could increase tumor metastasis through intra-tumoral hypoxia, resulting in increased tumor cell motility. Several studies have shown a parallel increase in HIF-1α expression during anti-VEGF therapies [124,127,128] associate with an increase in EMT changes which could account for an increased metastasis rate. 

The EMT process is a well-characterized mechanism essential in development and wound healing, and occurs in cancer metastasis [129]. During EMT, phenotypic changes essential for migration and invasion occur by the loss of apicobasal polarity, degradation of cell–cell junctions, cytoskeletal rearrangement, upregulation of mesenchymal markers, and loss of epithelial markers. Changes characterizing the EMT process comprise loss of epithelial proteins such as EPCAM, loss of adherens junction proteins such as E-cadherin, and concomitant activation of gene expression of mesenchymal proteins like vimentin, alpha-smooth muscle actin, and members of the Snail and Slug family [130,131]. These phenotypic changes have been observed after anti-angiogenic treatment, that cause tumors to acquire more angiogenic and invasive capacities, therefore promoting metastasis [86,132].

Overexpression of different HOX genes in a variety of tumor types is correlated with aggressive cellular behavior and the promotion of EMT. In addition to promoting the secretion of pro-angiogenic factors in tumor microenvironment, HOXB9 expression has been reported to promote cancer progression, EMT, and metastasis in different tumors. HOXB9 overexpression in breast cancer cell lines is associated with a transition from an epithelial phenotype into a more mesenchymal phenotype by reduction in E-cadherin expression levels and increasing of mesenchymal markers expression, including vimentin, snail, twist, and N-cadherin. These changes re-program breast cancer cells toward a more motile and invasive cells leading to metastatic nodules in lung in vivo [11]. Deregulation of HOXB9 expression facilitates migration and invasion of prostate cancer cells, whereas HOXB9 knockdown reverses the EMT process, inducing the expression of epithelial markers and the decrease in mesenchymal markers [68]. Enhanced migration and invasion caused by HOXB9 expression have been described also in colon cancer [49], endometrial cancer [59], and gliomas [51]. 

Recent studies have associated the EMT changes caused by HOXB9 expression with an upregulation of the TGF-β pathway. An overexpression of HOXB9 can activate the TGF-β pathway, leading to both an aggressive cellular phenotype and a switch towards an EMT phenotype [11].

The TGF-β signaling pathway plays an important role in driving cancer metastasis, essentially via induction of the EMT process [133]. In human cancers, cells with EMT characteristics have been detected particularly at the invasion front of the tumor, an area that is rich in stromal TGF-β and other cytokines that may cooperate in the induction of EMT. TGF-β promotes EMT by a combination of both Smad-dependent and Smad-independent mechanisms, requiring crosstalk between PI3K/AKT and Smad signaling proteins. Upon activation of the TGF-β and induction of the two zinc-finger transcription factors, Snail and Slug, a significant repression of E-cadherin is observed [134,135]. 

In this regard, it has been shown that HOXB9 could stimulate the migration and invasion of oral squamous carcinomas cells through activation of the TGF-β1/Smad2/Slug signaling pathway. Consistently, HOXB9 knockdown significantly reduces the expression of TGF-β1 and Smad2 phosphorylation, with concomitant suppression of Slug and Snail expression, resulting in a significant decrease in oral squamous cells motility [65]. HOXB9 overexpression significantly increases TGF-β1 expression and its downstream protein phospho-Smad2 also in glioma cells enhancing cells proliferation, migration, and invasion and accelerating the tumor growth in nude mice models. Conversely, HOXB9 knockdown inhibits the invasive behavior of glioma cells and downregulates the TGF-β1 signaling pathway [51]. These data have been supported by studies conducted in hepatocellular carcinomas, where elevated levels of HOXB9 were detected in both hepatocellular carcinomas tissue and hepatocellular cancer cell lines, and this was correlated with enhanced aggressive behavior of tumor cells and increased activation of the TGF-β1 pathway [61,62]. Therefore, HOXB9 might represent an important regulator of the TGF-β signaling. Analysis carried out in murine models resistant to anti-VEGF therapy have shown a positive correlation between HOXB9 expression and EMT phenotype. Silencing HOXB9 in the anti-VEGF-resistant xenograft mice significantly reverts their mesenchymal phenotype with a concomitant acquisition of sensitivity to anti-angiogenic therapy, resulting in prolonged survival [14]. 

All together, these data suggest that HOXB9 has an important function in regulating the more aggressive cancer cells phenotype acting at multiple levels to promote EMT and metastasis. 

### 4.3. HOXB9 in Modulating Stromal Cell in Tumor Microenvirment

The recruitment of various immune cell into the tumor microenvironment can be caused by increased hypoxia and upregulation of tumor secreted soluble factors such as PIGF, FGFs, ANG2 VEGF as well as cytokines such as G-CSF, C-C motif ligand 2 (CCL2), and SDF1. Cells recruited in the tumor microenvironment by these factors are bone marrow-derived cells (BMDCs) including myeloid-derived suppressor cells (MDSCs), monocytes and macrophages such as tumor-associated macrophages (TAMs) and cancer associated fibroblast (CAFs), which in turn can release additional angiogenic mediators sustaining tumor angiogenesis and an immunosuppressive tumor environment phenotype [136,137,138,139]. 

In particular, MDSC, also known as Gr1+ CD11b+ myeloid cells, consist of a heterogeneous population of myeloid cells with tumor promoting capacities [137,140]. In both, preclinical and clinical studies, an excessive recruitment of these myeloid cells, due to an increase production of G-CSF by the tumor, has been reported [141,142,143,144]. Notably, the infiltration of these myeloid population of cells is higher in anti-VEGF treatment refractory tumors and this infiltration contributes to tumor escape to anti-angiogenic treatments [145]. Furthermore, monocytes and macrophages can be recruited in the tumor microenvironment by different cytokines and chemokine released by tumors, including VEGF, CCL2, and macrophage colony stimulating factor (MCSF) [139]. Once recruited and infiltrated into the tumor, these macrophages, known as TAMs, can release multiple pro-angiogenic growth factors including TGF-β, VEGF, EGF, and chemokines, such as CCL2 and CXCL8, which contribute to the acquisition of resistance to anti-angiogenic therapy [146,147]. Adaptive resistance to anti-angiogenic therapy may also be mediated by an increased infiltration and activation of CAFs recruited by growth factors including TGF-β, PDGF, and FGF [148]. CAFs, in turn, produce several others growth factors that promote tumor growth, invasion, angiogenesis and immunosuppression [149,150]. 

Interestingly, *HOX* genes have also been implicated in directly regulating the phenotype of immune cells, thus suggesting a wider role for *HOX* genes in regulating the inflammatory environment [151]. HOXB9 expression has been associated with increased production of a number of angiogenic factors, such as VEGF, bFGF, Angptl2, TGF-β, IL-1, and IL-8, that could sustain the recruitment of CD11b+ myeloid cells in the tumor microenvironment [86]. A significantly greater infiltration of CD11b+ cells has been reported in a preclinical mouse model resistant to anti-VEGF treatment in HOXB9-higher tumor compared to their sensitive controls, suggesting that the proinflammatory factors overexpressed in this anti-angiogenic resistance tumor could sustain angiogenesis by inducing the recruitment of bone marrow–derived pro-angiogenic myeloid cells [14,86]. Inhibition of the proinflammatory signaling pathways or the HOXB9 downregulation in the anti-angiogenic resistant mouse model reverts CD11b+ cells tumor infiltration resulting in a more sensitive model to anti-angiogenic therapy and prolonging survival in vivo [14]. 

## 5. Conclusions

Angiogenesis is one of the hallmarks of cancer and the concept of targeting tumor angiogenesis represents a good cancer therapy strategy. Indeed, several new therapeutic drugs targeting the VEGF signaling pathway have been developed. Despite the promising expectations from preclinical studies, benefits derived from these therapies are limited. Treatment with anti-angiogenic agents may give rise to more resistant and aggressive tumors. To date, multiple mechanisms have been proposed for the resistance to anti-angiogenic therapies, but it mainly occurs thought the secretion of pro-angiogenic factors promoting tumor angiogenesis in a VEGF-independent manner and by the recruitment of myeloid cells, which in turn sustain inflammation. In this scenario, it would be important to select targeted therapies that block inflammatory pathways and stromal cell recruitment elicited during the anti-angiogenic treatment and also the identification of novel therapeutic targets in order to maximize clinical benefits.

In cancer *HOX* genes deregulation, arising through a variety of mechanisms, affects pathways that promote cell survival, proliferation and motility leading to the formation of more invasive tumor cells. *HOX* genes deregulation also induces upregulation of secreted angiogenic factors resulting in recruitment of myeloid cells. In this regard, recent findings emphasize the central role of the transcription factor HOXB9 in cancer development. Although not a transforming oncogene, HOXB9 has been shown to promote cancer progression and metastasis in different tumors via induction of cell motility and angiogenesis. HOXB9 could be a crucial transcription factor in sustaining tumor resistance to anti-VEGF treatment via modulation of the expression of alternative proinflammatory and pro-angiogenic secreted factors and via the recruitment of a subset of inflammatory immunosuppressive cells. Therefore, targeting the expression of HOXB9 could be a promising approach to modulate the tumor resistance to anti-angiogenic treatments. In addition, expression level of HOXB9 could be used as potential biomarker for selecting patients more likely to benefit from anti-angiogenic therapies.

## Figures and Tables

**Table 1:** FDA approved anti-angiogenic agents.

Drug Name	Molecular Target	Disease
Bevacizumab	VEGFA	Recurrent glioblastoma, metastatic colorectal cancer, metastatic non-squamous non-small cell lung, metastatic cervical cancer, metastatic renal cell carcinoma, recurrent epithelial ovarian cancer, fallopian tube cancer
Ramucirumab	VEGFR2	Advanced gastroesophageal junction adenocarcinoma and gastric adenocarcinoma, metastatic colorectal cancer, metastatic non-small cell lung cancer
Aflibercept	VEGFA, VEGFB, PIGF	Metastatic colorectal cancer
Sorafenib	VEGFRs, PDGFRs	Metastatic thyroid carcinoma, advanced renal cell carcinoma, advanced hepatocellular carcinoma
Sunitinib	VEGFRs, PDGFRs	Pancreatic neuroendocrine tumors, metastatic gastrointestinal stromal tumors, advanced renal cell carcinoma
Pazopanib	VEGFRs, PDGFRs, FGFRs	Advanced soft tissue carcinoma, advanced renal cell carcinoma
Axitinib	VEGFRs, PDGFRs,	Advanced renal cell carcinoma
Regorafenib	VEGFRs, PDGFRs, FGFRs	Advanced gastrointestinal stromal tumors, metastatic colorectal cancer, refractory hepatocellular carcinoma
Vandetanib	VEGFRs	Metastatic medullary thyroid cancer
Cabozantinib	VEGFRs, Tie2	Metastatic medullary thyroid cancer, refractory advanced renal carcinoma, refractory hepatocellular carcinoma
Lenvatinib	VEGFRs, PDGFRs, FGFRs	Recurrent and metastatic thyroid cancer, advanced hepatocellular carcinoma, advanced renal cell carcinoma, advanced endometrial carcinoma
Thalidomide	VEGFs, bFGF	Multiple myeloma
Lenalidomide	VEGFs, bFGF	Multiple myeloma, myelodysplastic syndromes, mantle cell lymphoma, follicular lymphoma, marginal zone lymphoma
Everolimus	mTOR	Advanced renal cell carcinoma, pancreatic neuroendocrine tumors, advanced breast cancer, subependymal giant cell astrocytoma

Current list of FDA-approved anti-angiogenic drugs for human cancer. Abbreviations: VEGFA, vascular endothelial growth factor A; VEGFB, vascular endothelial growth factor B; VEGFR2, vascular endothelial growth factor receptor 2; VEGFRs, vascular endothelial growth factor receptors; PIGF, placental growth factor; PDGFRs, platelet-derived growth factor receptors; FGFRs, fibroblast growth factor receptors; bFGF, basic fibroblast growth factors; mTOR, mammalian target of rapamycin, Tie2, tyrosine-protein kinase receptor.

**Table 2:** Overview of HOXB9 expression in cancer development and progression.

Tumor Type	Molecular Mechanism	Biological Effect	Clinical Observation	Reference
Breast cancer	It is the target gene of E2F1 transcription factor. Increased expression of VEGFA, bFGF, IL-8, and Angptl2.Enhanced EMT.	Produces highly vascularized tumors which developed lung metastases. It is involved in the DNA damage response and radiation resistance.	Overexpression is correlated with high tumor grade and poor survival.	[11,50,54,55,56]
Colorectal cancer	Increased expression of VEGFA, bFGF TGF-β and IL-8. Enhanced EMT.	Increases cell migration and invasion. The acetylated form decreases cancer progression.	Overexpression is correlated with distal metastasis and resistance to bavacizumab.	[14,49,57,58]
Endometrial cancer	Promoted E2F3 expression by direct targeting to its promoter.	Enhances cell migration and cancer progression.	High HOXB9 expression is associated with high histological grade and lymph node metastasis.	[59]
Gastric cancer	Suppress the phosphorylation of Akt and NF-κB activity. Induced MET.	Inhibits proliferation and migration of gastric cancer.	Decreased expression and overexpression is correlated with lymph node metastasis and poor survival.	[52,60]
Glioma	Activate the TGF-β1/Smad2 signaling.	Enhances cell proliferation, migration and sphere formation and increased tumorigenicity.	Overexpression is correlated with lymph node metastasis and poor survival.	[51]
Hepatocellular carcinoma	Enhanced EMT through the TGF-β1/Smad2 signaling. Regulated pro-angiogenic factors.	Promotes cell proliferation, migration, and invasion.	Overexpression is correlated with vascular invasion and poor prognosis.	[13,61,62]
Lung cancer	It is target gene of the WNT/TCF4 pathway. GalNAc-T14 induces expression of HOXB9 through Wnt signaling. PCFA-mediated HOXB9 acetylation.	Promotes cell invasion and mediates chemotactic invasion and colony outgrowth. The acetylated form decreases its capacity in promoting cell migration and tumor growth.	Overexpression is correlated with high tumor grade and poor prognosis.	[12,58,63,64]
Oral squamous carcinoma	Promoted EMT by TGF-β1/Smad2/Slug signaling.	Enhanced cell migration and invasion.	High HOXB9 levels are associated with high histological grade and shorter overall survival.	[65,66]
Ovarian and renal cancer	It is target gene of the miR-192.	Enhanced tumor angiogenesis.		[27]
Pancreatic cancer	Increased expression of VEGFA, bFGF, IL-8 and Angptl2. Enhanced EMT.	Promoted cell proliferation, migration, invasion, and sustained resistance to anti-VEGF inhibition. The acetylated form decreases tumor progression.	Overexpression is associated with shorter overall survival.	[14,67]
Prostate cancer	Enhanced EMT Regulated pro-angiogenic factors expression.	Promoted cell proliferation, migration, invasion, and angiogenesis ability.	Overexpression is correlated with vascular invasion and poor prognosis.	[68]

Summary of studies evaluating the role of HOXB9 in solid tumor progression.

## References

[1] Tortora G., Melisi D., Ciardiello F. (2004). Angiogenesis: A target for cancer therapy. Curr. Pharm. Des..

[2] Ferrara N. (2002). VEGF and the quest for tumour angiogenesis factors. Nat. Rev. Cancer.

[3] Ferrara N., Adamis A.P. (2016). Ten years of anti-vascular endothelial growth factor therapy. Nat. Rev. Drug Discov..

[4] Ferrara N., Hillan K.J., Gerber H.-P., Novotny W. (2004). Discovery and development of bevacizumab, an anti-VEGF antibody for treating cancer. Nat. Rev. Drug Discov..

[5] Hurwitz H., Fehrenbacher L., Novotny W., Cartwright T., Hainsworth J., Heim W., Berlin J., Baron A., Griffing S., Holmgren E. (2004). Bevacizumab plus irinotecan, fluorouracil, and leucovorin for metastatic colorectal cancer. N. Engl. J. Med..

[6] Wilke H., Muro K., Van Cutsem E., Oh S.-C., Bodoky G., Shimada Y., Hironaka S., Sugimoto N., Lipatov O., Kim T.-Y. (2014). Ramucirumab plus paclitaxel versus placebo plus paclitaxel in patients with previously treated advanced gastric or gastro-oesophageal junction adenocarcinoma (RAINBOW): A double-blind, randomised phase 3 trial. Lancet Oncol..

[7] Garon E.B., Ciuleanu T.-E., Arrieta O., Prabhash K., Syrigos K.N., Goksel T., Park K., Gorbunova V., Kowalyszyn R.D., Pikiel J. (2014). Ramucirumab plus docetaxel versus placebo plus docetaxel for second-line treatment of stage IV non-small-cell lung cancer after disease progression on platinum-based therapy (REVEL): A multicentre, double-blind, randomised phase 3 trial. Lancet.

[8] Ciombor K.K., Berlin J., Chan E. (2013). Aflibercept. Clin. Cancer Res..

[9] Ebos J.M., Kerbel R.S. (2011). Antiangiogenic therapy: Impact on invasion, disease progression, and metastasis. Nat. Rev. Clin. Oncol..

[10] Van Beijnum J.R., Nowak-Sliwinska P., Huijbers E.J., Thijssen V.L., Griffioen A.W. (2015). The great escape; the hallmarks of resistance to antiangiogenic therapy. Pharmacol. Rev..

[11] Hayashida T., Takahashi F., Chiba N., Brachtel E., Takahashi M., Godin-Heymann N., Gross K.W., Vivanco M.d.M., Wijendran V., Shioda T. (2010). HOXB9, a gene overexpressed in breast cancer, promotes tumorigenicity and lung metastasis. Proc. Natl. Acad. Sci. USA.

[12] Zhan J., Wang P., Niu M., Wang Y., Zhu X., Guo Y., Zhang H. (2015). High expression of transcriptional factor HoxB9 predicts poor prognosis in patients with lung adenocarcinoma. Histopathology.

[13] Chiba N., Ozawa Y., Hikita K., Okihara M., Sano T., Tomita K., Takano K., Kawachi S. (2017). Increased expression of HOXB9 in hepatocellular carcinoma predicts poor overall survival but a beneficial response to sorafenib. Oncol. Rep..

[14] Carbone C., Piro G., Simionato F., Ligorio F., Cremolini C., Loupakis F., Alì G., Rossini D., Merz V., Santoro R. (2017). Homeobox B9 mediates resistance to anti-VEGF therapy in colorectal cancer patients. Clin. Cancer Res..

[15] Bridges C.B. (1921). Current maps of the location of the mutant genes of Drosophila melanogaster. Proc. Natl. Acad. Sci. USA.

[16] Lewis E.B. (1978). A gene complex controlling segmentation in Drosophila. Genes, Development and Cancer.

[17] Scott M.P. (1992). Vertebrate homeobox gene nomenclature. Cell.

[18] Levine M., Hoey T. (1988). Homeobox proteins as sequence-specific transcription factors. Cell.

[19] McGinnis W., Krumlauf R. (1992). Homeobox genes and axial patterning. Cell.

[20] Mann R.S., Lelli K.M., Joshi R. (2009). Hox specificity: Unique roles for cofactors and collaborators. Curr. Top. Dev. Biol..

[21] Iimura T., Pourquié O. (2007). Hox genes in time and space during vertebrate body formation. Dev. Growth Differ..

[22] Horan G.S., Kovàcs E.N., Behringer R.R., Featherstone M.S. (1995). Mutations in paralogous Hox genes result in overlapping homeotic transformations of the axial skeleton: Evidence for unique and redundant function. Dev. Biol..

[23] Greer J.M., Puetz J., Thomas K.R., Capecchi M.R. (2000). Maintenance of functional equivalence during paralogous Hox gene evolution. Nature.

[24] Mansour M.A., Senga T. (2017). HOXD8 exerts a tumor-suppressing role in colorectal cancer as an apoptotic inducer. Int. J. Biochem. Cell Biol..

[25] De Kumar B., Parker H.J., Paulson A., Parrish M.E., Zeitlinger J., Krumlauf R. (2017). Hoxa1 targets signaling pathways during neural differentiation of ES cells and mouse embryogenesis. Dev. Biol..

[26] Tsuboi M., Taniuchi K., Shimizu T., Saito M., Saibara T. (2017). The transcription factor HOXB7 regulates ERK kinase activity and thereby stimulates the motility and invasiveness of pancreatic cancer cells. J. Biol. Chem..

[27] Wu S.Y., Rupaimoole R., Shen F., Pradeep S., Pecot C.V., Ivan C., Nagaraja A.S., Gharpure K.M., Pham E., Hatakeyama H. (2016). A miR-192-EGR1-HOXB9 regulatory network controls the angiogenic switch in cancer. Nat. Commun..

[28] Boudreau N., Andrews C., Srebrow A., Ravanpay A., Cheresh D.A. (1997). Induction of the angiogenic phenotype by Hox D3. J. Cell Biol..

[29] Brooks P.C., Clark R.A., Cheresh D.A. (1994). Requirement of vascular integrin alpha v beta 3 for angiogenesis. Science.

[30] Chisaka O., Kameda Y. (2005). Hoxa3 regulates the proliferation and differentiation of the third pharyngeal arch mesenchyme in mice. Cell Tissue Res..

[31] Myers C., Charboneau A., Boudreau N. (2000). Homeobox B3 promotes capillary morphogenesis and angiogenesis. J. Cell Biol..

[32] Bruhl T., Urbich C., Aicher D., Acker-Palmer A., Zeiher A.M., Dimmeler S. (2004). Homeobox A9 transcriptionally regulates the EphB4 receptor to modulate endothelial cell migration and tube formation. Circ. Res..

[33] Rössig L., Urbich C., Brühl T., Dernbach E., Heeschen C., Chavakis E., Sasaki K.-I., Aicher D., Diehl F., Seeger F. (2005). Histone deacetylase activity is essential for the expression of HoxA9 and for endothelial commitment of progenitor cells. J. Exp. Med..

[34] Wu Y., Moser M., Bautch V.L., Patterson C. (2003). HoxB5 is an upstream transcriptional switch for differentiation of the vascular endothelium from precursor cells. Mol. Cell. Biol..

[35] Carè A., Silvani A., Meccia E., Mattia G., Stoppacciaro A., Parmiani G., Peschle C., Colombo M.P. (1996). HOXB7 constitutively activates basic fibroblast growth factor in melanomas. Mol. Cell. Biol..

[36] Carè A., Felicetti F., Meccia E., Bottero L., Parenza M., Stoppacciaro A., Peschle C., Colombo M.P. (2001). HOXB7: A key factor for tumor-associated angiogenic switch. Cancer Res..

[37] Tan Z., Chen K., Wu W., Zhou Y., Zhu J., Wu G., Cao L., Zhang X., Guan H., Yang Y. (2018). Overexpression of HOXC10 promotes angiogenesis in human glioma via interaction with PRMT5 and upregulation of VEGFA expression. Theranostics.

[38] Rhoads K., Arderiu G., Charboneau A., Hansen S.L., Hoffman W., Boudreau N. (2005). A role for Hox A5 in regulating angiogenesis and vascular patterning. Lymphat. Res. Biol..

[39] Arderiu G., Cuevas I., Chen A., Carrio M., East L., Boudreau N.J. (2007). HoxA5 stabilizes adherens junctions via increased Akt1. Cell Adhes. Migr..

[40] Myers C., Charboneau A., Cheung I., Hanks D., Boudreau N. (2002). Sustained expression of homeobox D10 inhibits angiogenesis. Am. J. Pathol..

[41] Carrio M., Arderiu G., Myers C., Boudreau N.J. (2005). Homeobox D10 induces phenotypic reversion of breast tumor cells in a three-dimensional culture model. Cancer Res..

[42] Chen A., Cuevas I., Kenny P.A., Miyake H., Mace K., Ghajar C., Boudreau A., Bissell M., Boudreau N. (2009). Endothelial cell migration and vascular endothelial growth factor expression are the result of loss of breast tissue polarity. Cancer Res..

[43] Fromental-Ramain C., Warot X., Lakkaraju S., Favier B., Haack H., Birling C., Dierich A., Chambon P. (1996). Specific and redundant functions of the paralogous Hoxa-9 and Hoxd-9 genes in forelimb and axial skeleton patterning. Development.

[44] Xu B., Wellik D.M. (2011). Axial Hox9 activity establishes the posterior field in the developing forelimb. Proc. Natl. Acad. Sci. USA.

[45] Chen F., Capecchi M.R. (1999). Paralogous mouse Hox genes, Hoxa9, Hoxb9, and Hoxd9, function together to control development of the mammary gland in response to pregnancy. Proc. Natl. Acad. Sci. USA.

[46] Magli M.C., Largman C., Lawrence H.J. (1997). Effects of HOX homeobox genes in blood cell differentiation. J. Cell. Physiol..

[47] Shah N., Sukumar S. (2010). The Hox genes and their roles in oncogenesis. Nat. Rev. Cancer.

[48] Bhatlekar S., Fields J.Z., Boman B.M. (2014). HOX genes and their role in the development of human cancers. J. Mol. Med..

[49] Huang K., Yuan R., Wang K., Hu J., Huang Z., Yan C., Shen W., Shao J. (2014). Overexpression of HOXB9 promotes metastasis and indicates poor prognosis in colon cancer. Chin. J. Cancer Res..

[50] Seki H., Hayashida T., Jinno H., Hirose S., Sakata M., Takahashi M., Maheswaran S., Mukai M., Kitagawa Y. (2012). HOXB9 expression promoting tumor cell proliferation and angiogenesis is associated with clinical outcomes in breast cancer patients. Ann. Surg. Oncol..

[51] Fang L., Xu Y., Zou L. (2014). Overexpressed homeobox B9 regulates oncogenic activities by transforming growh factor-β1 in gliomas. Biochem. Biophys. Res. Commun..

[52] Kato F., Wada N., Hayashida T., Fukuda K., Nakamura R., Takahashi T., Kawakubo H., Takeuchi H., Kitagawa Y. (2019). Experimental and clinicopathological analysis of HOXB9 in gastric cancer. Oncol. Lett..

[53] Sha S., Gu Y., Xu B., Hu H., Yang Y., Kong X., Wu K. (2013). Decreased expression of HOXB9 is related to poor overall survival in patients with gastric carcinoma. Dig. Liver Dis..

[54] Shrestha B., Ansari K.I., Bhan A., Kasiri S., Hussain I., Mandal S.S. (2012). Homeodomain-containing protein HOXB 9 regulates expression of growth and angiogenic factors, facilitates tumor growth in vitro and is overexpressed in breast cancer tissue. FEBS J..

[55] Chiba N., Comaills V., Shiotani B., Takahashi F., Shimada T., Tajima K., Winokur D., Hayashida T., Willers H., Brachtel E. (2012). Homeobox B9 induces epithelial-to-mesenchymal transition-associated radioresistance by accelerating DNA damage responses. Proc. Natl. Acad. Sci. USA.

[56] Zhussupova A., Hayashida T., Takahashi M., Miyao K., Okazaki H., Jinno H., Kitagawa Y. (2014). An E2F1-HOXB9 transcriptional circuit is associated with breast cancer progression. PLoS ONE.

[57] Hoshino Y., Hayashida T., Hirata A., Takahashi H., Chiba N., Ohmura M., Wakui M., Jinno H., Hasegawa H., Maheswaran S. (2014). Bevacizumab terminates homeobox B9-induced tumor proliferation by silencing microenvironmental communication. Mol. Cancer.

[58] Wan J., Xu W., Zhan J., Ma J., Li X., Xie Y., Wang J., Zhu W.-G., Luo J., Zhang H. (2016). PCAF-mediated acetylation of transcriptional factor HOXB9 suppresses lung adenocarcinoma progression by targeting oncogenic protein JMJD6. Nucleic Acids Res..

[59] Wan J., Liu H., Feng Q., Liu J., Ming L. (2018). HOXB9 promotes endometrial cancer progression by targeting E2F3. Cell Death Dis..

[60] Zhang L., Wu Q., He C., Liang D., Yi Q., Shi J., Wan B., Yang R., Li L., Sha S. (2019). HOXB9 inhibits proliferation in gastric carcinoma cells via suppression of phosphorylated-Akt and NF-κB-dependent Snail expression. Dig. Liver Dis..

[61] Sha L., Dong L., Lv L., Bai L., Ji X. (2015). HOXB9 promotes epithelial-to-mesenchymal transition via transforming growth factor-β1 pathway in hepatocellular carcinoma cells. Clin. Exp. Med..

[62] Li F., Dong L., Xing R., Wang L., Luan F., Yao C., Ji X., Bai L. (2014). Homeobox B9 is overexpressed in hepatocellular carcinomas and promotes tumor cell proliferation both in vitro and in vivo. Biochem. Biophys. Res. Commun..

[63] Nguyen D.X., Chiang A.C., Zhang X.H.-F., Kim J.Y., Kris M.G., Ladanyi M., Gerald W.L., Massagué J. (2009). WNT/TCF signaling through LEF1 and HOXB9 mediates lung adenocarcinoma metastasis. Cell.

[64] Kwon O.-S., Oh E., Park J.-R., Lee J.-S., Bae G.-Y., Koo J.-H., Kim H., Choi Y.-L., Choi Y.S., Kim J. (2015). GalNAc-T14 promotes metastasis through Wnt dependent HOXB9 expression in lung adenocarcinoma. Oncotarget.

[65] Xue M., Zhu F.-Y., Chen L., Wang K. (2017). HoxB9 promotes the migration and invasion via TGF-β1/Smad2/Slug signaling pathway in oral squamous cell carcinoma. Am. J. Transl. Res..

[66] Sun C., Han C., Wang P., Jin Y., Sun Y., Qu L. (2017). HOXB9 expression correlates with histological grade and prognosis in LSCC. Biomed Res. Int..

[67] Sun X., Song J., Zhang J., Zhan J., Fang W., Zhang H. (2020). Acetylated HOXB9 at lysine 27 is of differential diagnostic value in patients with pancreatic ductal adenocarcinoma. Front. Med..

[68] Xu H., Wu S., Shen X., Wu D., Qin Z., Wang H., Chen X., Sun X. (2020). Silencing of HOXB9 suppresses cellular proliferation, angiogenesis, migration and invasion of prostate cancer cells. J. Biosci..

[69] Hatzis P., van der Flier L.G., van Driel M.A., Guryev V., Nielsen F., Denissov S., Nijman I.J., Koster J., Santo E.E., Welboren W. (2008). Genome-wide pattern of TCF7L2/TCF4 chromatin occupancy in colorectal cancer cells. Mol. Cell. Biol..

[70] Taylor H.S. (2000). The role of HOX genes in human implantation. Hum. Reprod. Update.

[71] Ansari K.I., Shrestha B., Hussain I., Kasiri S., Mandal S.S. (2011). Histone methylases MLL1 and MLL3 coordinate with estrogen receptors in estrogen-mediated HOXB9 expression. Biochemistry.

[72] Song J., Wang T., Xu W., Wang P., Wan J., Wang Y., Zhan J., Zhang H. (2018). HOXB9 acetylation at K27 is responsible for its suppression of colon cancer progression. Cancer Lett..

[73] Miller K.D., Sweeney C.J., Sledge G.W. (2005). Can tumor angiogenesis be inhibited without resistance. Mechanisms of Angiogenesis.

[74] Ruegg C., Mutter N. (2007). Anti-angiogenic therapies in cancer: Achievements and open questions. Bull. Cancer.

[75] Bergers G., Hanahan D. (2008). Modes of resistance to anti-angiogenic therapy. Nat. Rev. Cancer.

[76] Zhong H., De Marzo A.M., Laughner E., Lim M., Hilton D.A., Zagzag D., Buechler P., Isaacs W.B., Semenza G.L., Simons J.W. (1999). Overexpression of hypoxia-inducible factor 1α in common human cancers and their metastases. Cancer Res..

[77] Hayashida T., Jinno H., Seki H., Takahashi M., Sakata M., Hirose S., Mukai M., Kitagawa Y. (2011). The relationship of HOXB9 expression promoting tumor cell proliferation and angiogenesis to clinical outcomes of patients with breast cancer. J. Clin. Oncol..

[78] Jain R.K. (2003). Molecular regulation of vessel maturation. Nat. Med..

[79] Ornitz D.M., Itoh N. (2015). The Fibroblast Growth Factor signaling pathway. Wiley Interdiscip Rev. Dev. Biol..

[80] Teven C.M., Farina E.M., Rivas J., Reid R.R. (2014). Fibroblast growth factor (FGF) signaling in development and skeletal diseases. Genes Dis..

[81] Eswarakumar V., Lax I., Schlessinger J. (2005). Cellular signaling by fibroblast growth factor receptors. Cytokine Growth Factor Rev..

[82] Presta M., Dell’Era P., Mitola S., Moroni E., Ronca R., Rusnati M. (2005). Fibroblast growth factor/fibroblast growth factor receptor system in angiogenesis. Cytokine Growth Factor Rev..

[83] Casanovas O., Hicklin D.J., Bergers G., Hanahan D. (2005). Drug resistance by evasion of antiangiogenic targeting of VEGF signaling in late-stage pancreatic islet tumors. Cancer Cell.

[84] Kopetz S., Hoff P.M., Morris J.S., Wolff R.A., Eng C., Glover K.Y., Adinin R., Overman M.J., Valero V., Wen S. (2010). Phase II trial of infusional fluorouracil, irinotecan, and bevacizumab for metastatic colorectal cancer: Efficacy and circulating angiogenic biomarkers associated with therapeutic resistance. J. Clin. Oncol..

[85] Batchelor T.T., Sorensen A.G., di Tomaso E., Zhang W.-T., Duda D.G., Cohen K.S., Kozak K.R., Cahill D.P., Chen P.-J., Zhu M. (2007). AZD2171, a pan-VEGF receptor tyrosine kinase inhibitor, normalizes tumor vasculature and alleviates edema in glioblastoma patients. Cancer Cell.

[86] Carbone C., Moccia T., Zhu C., Paradiso G., Budillon A., Chiao P.J., Abbruzzese J.L., Melisi D. (2011). Anti-VEGF treatment-resistant pancreatic cancers secrete proinflammatory factors that contribute to malignant progression by inducing an EMT cell phenotype. Clin. Cancer Res..

[87] Alfaro C., Sanmamed M.F., Rodríguez-Ruiz M.E., Teijeira Á., Oñate C., González Á., Ponz M., Schalper K.A., Pérez-Gracia J.L., Melero I. (2017). Interleukin-8 in cancer pathogenesis, treatment and follow-up. Cancer Treat. Rev..

[88] Merz V., Zecchetto C., Santoro R., Simionato F., Sabbadini F., Mangiameli D., Piro G., Cavaliere A., Deiana M., Valenti M.T. (2020). Plasma IL8 Is a Biomarker for TAK1 Activation and Predicts Resistance to Nanoliposomal Irinotecan in Patients with Gemcitabine-Refractory Pancreatic Cancer. Clin. Cancer Res..

[89] Gyanchandani R., Sano D., Alves M.V.O., Klein J.D., Knapick B.A., Oh S., Myers J.N., Kim S. (2013). Interleukin-8 as a modulator of response to bevacizumab in preclinical models of head and neck squamous cell carcinoma. Oral Oncol..

[90] Huang D., Ding Y., Zhou M., Rini B.I., Petillo D., Qian C.-N., Kahnoski R., Futreal P.A., Furge K.A., Teh B.T. (2010). Interleukin-8 mediates resistance to antiangiogenic agent sunitinib in renal cell carcinoma. Cancer Res..

[91] Batchelor T.T., Gerstner E.R., Emblem K.E., Duda D.G., Kalpathy-Cramer J., Snuderl M., Ancukiewicz M., Polaskova P., Pinho M.C., Jennings D. (2013). Improved tumor oxygenation and survival in glioblastoma patients who show increased blood perfusion after cediranib and chemoradiation. Proc. Natl. Acad. Sci. USA.

[92] Voronov E., Carmi Y., Apte R.N. (2014). The role IL-1 in tumor-mediated angiogenesis. Front. Physiol..

[93] Acuner Ozbabacan S.E., Gursoy A., Nussinov R., Keskin O. (2014). The structural pathway of interleukin 1 (IL-1) initiated signaling reveals mechanisms of oncogenic mutations and SNPs in inflammation and cancer. PLoS Comput. Biol..

[94] Carbone C., Melisi D. (2012). NF-kappaB as a target for pancreatic cancer therapy. Expert Opin. Targets.

[95] Melisi D., Chiao P.J. (2007). NF-kappa B as a target for cancer therapy. Expert Opin. Targets.

[96] Melisi D., Niu J., Chang Z., Xia Q., Peng B., Ishiyama S., Evans D.B., Chiao P.J. (2009). Secreted interleukin-1alpha induces a metastatic phenotype in pancreatic cancer by sustaining a constitutive activation of nuclear factor-kappaB. Mol. Cancer Res..

[97] Zhuang Z., Ju H.Q., Aguilar M., Gocho T., Li H., Iida T., Lee H., Fan X., Zhou H., Ling J. (2016). IL1 Receptor Antagonist Inhibits Pancreatic Cancer Growth by Abrogating NF-kappaB Activation. Clin. Cancer Res..

[98] Saijo Y., Tanaka M., Miki M., Usui K., Suzuki T., Maemondo M., Hong X., Tazawa R., Kikuchi T., Matsushima K. (2002). Proinflammatory cytokine IL-1β promotes tumor growth of Lewis lung carcinoma by induction of angiogenic factors: In vivo analysis of tumor-stromal interaction. J. Immunol..

[99] Carbone C., Tamburrino A., Piro G., Boschi F., Cataldo I., Zanotto M., Mina M.M., Zanini S., Sbarbati A., Scarpa A. (2016). Combined inhibition of IL1, CXCR1/2, and TGFbeta signaling pathways modulates in-vivo resistance to anti-VEGF treatment. Anticancer Drugs.

[100] Goumans M.-J., Liu Z., Ten Dijke P. (2009). TGF-β signaling in vascular biology and dysfunction. Cell Res..

[101] Massagué J. (2008). TGFβ in cancer. Cell.

[102] Melisi D., Garcia-Carbonero R., Macarulla T., Pezet D., Deplanque G., Fuchs M., Trojan J., Kozloff M., Simionato F., Cleverly A. (2019). TGFbeta receptor inhibitor galunisertib is linked to inflammation- and remodeling-related proteins in patients with pancreatic cancer. Cancer Chemother. Pharmacol..

[103] Melisi D., Garcia-Carbonero R., Macarulla T., Pezet D., Deplanque G., Fuchs M., Trojan J., Oettle H., Kozloff M., Cleverly A. (2018). Galunisertib plus gemcitabine vs. gemcitabine for first-line treatment of patients with unresectable pancreatic cancer. Br. J. Cancer.

[104] Melisi D., Hollebecque A., Oh do Y., Calvo E., Varghese A., Borazanci E., Macarulla T., Simionato F., Park O.J., Bendell J. (2019). A Phase 1b Dose-Escalation and Cohort-Expansion Study of Safety and Activity of the Transforming Growth Factor (TGF) β Receptor I Kinase Inhibitor Galunisertib Plus the Anti-PD-L1 Antibody Durvalumab in Metastatic Pancreatic Cancer. J. Clin. Oncol..

[105] Melisi D., Ishiyama S., Sclabas G.M., Fleming J.B., Xia Q., Tortora G., Abbruzzese J.L., Chiao P.J. (2008). LY2109761, a novel transforming growth factor beta receptor type I and type II dual inhibitor, as a therapeutic approach to suppressing pancreatic cancer metastasis. Mol. Cancer.

[106] Melisi D., Xia Q., Paradiso G., Ling J., Moccia T., Carbone C., Budillon A., Abbruzzese J.L., Chiao P.J. (2011). Modulation of pancreatic cancer chemoresistance by inhibition of TAK1. J. Natl. Cancer Inst..

[107] Piro G., Giacopuzzi S., Bencivenga M., Carbone C., Verlato G., Frizziero M., Zanotto M., Mina M.M., Merz V., Santoro R. (2015). TAK1-regulated expression of BIRC3 predicts resistance to preoperative chemoradiotherapy in oesophageal adenocarcinoma patients. Br. J. Cancer.

[108] Santoro R., Carbone C., Piro G., Chiao P.J., Melisi D. (2017). TAK-ing aim at chemoresistance: The emerging role of MAP3K7 as a target for cancer therapy. Drug Resist. Updates.

[109] Santoro R., Zanotto M., Simionato F., Zecchetto C., Merz V., Cavallini C., Piro G., Sabbadini F., Boschi F., Scarpa A. (2019). Modulating TAK1 expression inhibits YAP and TAZ oncogenic functions in pancreatic cancer. Mol. Cancer.

[110] Cunha S.I., Pietras K. (2011). ALK1 as an emerging target for antiangiogenic therapy of cancer. Blood.

[111] Bertolino P., Deckers M., Lebrin F., ten Dijke P. (2005). Transforming growth factor-β signal transduction in angiogenesis and vascular disorders. Chest.

[112] Oh S.P., Seki T., Goss K.A., Imamura T., Yi Y., Donahoe P.K., Li L., Miyazono K., ten Dijke P., Kim S. (2000). Activin receptor-like kinase 1 modulates transforming growth factor-β1 signaling in the regulation of angiogenesis. Proc. Natl. Acad. Sci. USA.

[113] Chen X.-L., Chen Z.-Q., Zhu S.-L., Liu T.-W., Wen Y., Su Y.-S., Xi X.-J., Hu Y., Lian L., Liu F.-B. (2017). Prognostic value of transforming growth factor-beta in patients with colorectal cancer who undergo surgery: A meta-analysis. BMC Cancer.

[114] Dave H., Shah M., Trivedi S., Shukla S. (2012). Prognostic utility of circulating transforming growth factor beta 1 in breast cancer patients. Int. J. Biol. Markers.

[115] Park S.Y., Piao Y., Jeong K.J., Dong J., de Groot J.F. (2016). Periostin (POSTN) regulates tumor resistance to antiangiogenic therapy in glioma models. Mol. Cancer Ther..

[116] Bockhorn M., Tsuzuki Y., Xu L., Frilling A., Broelsch C.E., Fukumura D. (2003). Differential vascular and transcriptional responses to anti-vascular endothelial growth factor antibody in orthotopic human pancreatic cancer xenografts. Clin. Cancer Res..

[117] Carbone C., Piro G., Merz V., Simionato F., Santoro R., Zecchetto C., Tortora G., Melisi D. (2018). Angiopoietin-Like Proteins in Angiogenesis, Inflammation and Cancer. Int. J. Mol. Sci..

[118] Aoi J., Endo M., Kadomatsu T., Miyata K., Nakano M., Horiguchi H., Ogata A., Odagiri H., Yano M., Araki K. (2011). Angiopoietin-like protein 2 is an important facilitator of inflammatory carcinogenesis and metastasis. Cancer Res..

[119] Endo M., Nakano M., Kadomatsu T., Fukuhara S., Kuroda H., Mikami S., Hato T., Aoi J., Horiguchi H., Miyata K. (2012). Tumor Cell-Derived Angiopoietin-like Protein ANGPTL2 Is a Critical Driver of Metastasis. Cancer Res..

[120] Carbone C., Piro G., Fassan M., Tamburrino A., Mina M.M., Zanotto M., Chiao P.J., Bassi C., Scarpa A., Tortora G. (2015). An angiopoietin-like protein 2 autocrine signaling promotes EMT during pancreatic ductal carcinogenesis. Oncotarget.

[121] Hato T., Tabata M., Oike Y. (2008). The role of angiopoietin-like proteins in angiogenesis and metabolism. Trends Cardiovasc. Med..

[122] Kubota Y., Oike Y., Satoh S., Tabata Y., Niikura Y., Morisada T., Akao M., Urano T., Ito Y., Miyamoto T. (2005). Cooperative interaction of Angiopoietin-like proteins 1 and 2 in zebrafish vascular development. Proc. Natl. Acad. Sci. USA.

[123] Ebos J.M., Lee C.R., Cruz-Munoz W., Bjarnason G.A., Christensen J.G., Kerbel R.S. (2009). Accelerated metastasis after short-term treatment with a potent inhibitor of tumor angiogenesis. Cancer Cell.

[124] Pàez-Ribes M., Allen E., Hudock J., Takeda T., Okuyama H., Viñals F., Inoue M., Bergers G., Hanahan D., Casanovas O. (2009). Antiangiogenic therapy elicits malignant progression of tumors to increased local invasion and distant metastasis. Cancer Cell.

[125] Chung A.S., Kowanetz M., Wu X., Zhuang G., Ngu H., Finkle D., Komuves L., Peale F., Ferrara N. (2012). Differential drug class-specific metastatic effects following treatment with a panel of angiogenesis inhibitors. J. Pathol..

[126] Welti J.C., Powles T., Foo S., Gourlaouen M., Preece N., Foster J., Frentzas S., Bird D., Sharpe K., Van Weverwijk A. (2012). Contrasting effects of sunitinib within in vivo models of metastasis. Angiogenesis.

[127] Cooke V.G., LeBleu V.S., Keskin D., Khan Z., O’Connell J.T., Teng Y., Duncan M.B., Xie L., Maeda G., Vong S. (2012). Pericyte depletion results in hypoxia-associated epithelial-to-mesenchymal transition and metastasis mediated by met signaling pathway. Cancer Cell.

[128] Rovida A., Castiglioni V., Decio A., Scarlato V., Scanziani E., Giavazzi R., Cesca M. (2013). Chemotherapy counteracts metastatic dissemination induced by antiangiogenic treatment in mice. Mol. Cancer Ther..

[129] Gaianigo N., Melisi D., Carbone C. (2017). EMT and Treatment Resistance in Pancreatic Cancer. Cancers.

[130] Aiello N.M., Kang Y. (2019). Context-dependent EMT programs in cancer metastasis. J. Exp. Med..

[131] Micalizzi D.S., Farabaugh S.M., Ford H.L. (2010). Epithelial-mesenchymal transition in cancer: Parallels between normal development and tumor progression. J. Mammary Gland Biol. Neoplasia.

[132] Jung H.-Y., Fattet L., Yang J. (2015). Molecular pathways: Linking tumor microenvironment to epithelial–mesenchymal transition in metastasis. Clin. Cancer Res..

[133] Padua D., Massagué J. (2009). Roles of TGFβ in metastasis. Cell Res..

[134] Cho H.J., Baek K.E., Saika S., Jeong M.-J., Yoo J. (2007). Snail is required for transforming growth factor-β-induced epithelial–mesenchymal transition by activating PI3 kinase/Akt signal pathway. Biochem. Biophys. Res. Commun..

[135] Naber H.P., Drabsch Y., Snaar-Jagalska B.E., ten Dijke P., van Laar T. (2013). Snail and Slug, key regulators of TGF-β-induced EMT, are sufficient for the induction of single-cell invasion. Biochem. Biophys. Res. Commun..

[136] Grunewald M., Avraham I., Dor Y., Bachar-Lustig E., Itin A., Yung S., Chimenti S., Landsman L., Abramovitch R., Keshet E. (2006). VEGF-induced adult neovascularization: Recruitment, retention, and role of accessory cells. Cell.

[137] Crawford Y., Ferrara N. (2009). Tumor and stromal pathways mediating refractoriness/resistance to anti-angiogenic therapies. Trends Pharmacol. Sci..

[138] Solinas G., Germano G., Mantovani A., Allavena P. (2009). Tumor-associated macrophages (TAM) as major players of the cancer-related inflammation. J. Leukoc. Biol..

[139] Capece D., Fischietti M., Verzella D., Gaggiano A., Cicciarelli G., Tessitore A., Zazzeroni F., Alesse E. (2013). The inflammatory microenvironment in hepatocellular carcinoma: A pivotal role for tumor-associated macrophages. Biomed Res. Int..

[140] Shojaei F., Ferrara N. (2008). Refractoriness to antivascular endothelial growth factor treatment: Role of myeloid cells. Cancer Res..

[141] Yang L., DeBusk L.M., Fukuda K., Fingleton B., Green-Jarvis B., Shyr Y., Matrisian L.M., Carbone D.P., Lin P.C. (2004). Expansion of myeloid immune suppressor Gr+ CD11b+ cells in tumor-bearing host directly promotes tumor angiogenesis. Cancer Cell.

[142] Marigo I., Dolcetti L., Serafini P., Zanovello P., Bronte V. (2008). Tumor-induced tolerance and immune suppression by myeloid derived suppressor cells. Immunol. Rev..

[143] Diaz-Montero C.M., Salem M.L., Nishimura M.I., Garrett-Mayer E., Cole D.J., Montero A.J. (2009). Increased circulating myeloid-derived suppressor cells correlate with clinical cancer stage, metastatic tumor burden, and doxorubicin–cyclophosphamide chemotherapy. Cancer Immunol. Immunother..

[144] Shojaei F., Wu X., Qu X., Kowanetz M., Yu L., Tan M., Meng Y.G., Ferrara N. (2009). G-CSF-initiated myeloid cell mobilization and angiogenesis mediate tumor refractoriness to anti-VEGF therapy in mouse models. Proc. Natl. Acad. Sci. USA.

[145] Shojaei F., Wu X., Malik A.K., Zhong C., Baldwin M.E., Schanz S., Fuh G., Gerber H.-P., Ferrara N. (2007). Tumor refractoriness to anti-VEGF treatment is mediated by CD11b+ Gr1+ myeloid cells. Nat. Biotechnol..

[146] Murdoch C., Muthana M., Coffelt S.B., Lewis C.E. (2008). The role of myeloid cells in the promotion of tumour angiogenesis. Nat. Rev. Cancer.

[147] Lin E.Y., Li J.-F., Gnatovskiy L., Deng Y., Zhu L., Grzesik D.A., Qian H., Xue X.-N., Pollard J.W. (2006). Macrophages regulate the angiogenic switch in a mouse model of breast cancer. Cancer Res..

[148] Turner N., Grose R. (2010). Fibroblast growth factor signalling: From development to cancer. Nat. Rev. Cancer.

[149] Bhowmick N.A., Neilson E.G., Moses H.L. (2004). Stromal fibroblasts in cancer initiation and progression. Nature.

[150] Clarke J.M., Hurwitz H.I. (2013). Understanding and targeting resistance to anti-angiogenic therapies. J. Gastrointest. Oncol..

[151] Mahdipour E., Mace K.A. (2011). Hox transcription factor regulation of adult bone-marrow-derived cell behaviour during tissue repair and regeneration. Expert Opin. Biol. Ther..

